# Knowledge and attitude of surgical patients and their families toward anesthesia

**DOI:** 10.3389/fmed.2024.1371785

**Published:** 2024-05-22

**Authors:** Jie Wang, Shuai Wang, Ruifeng Zeng

**Affiliations:** ^1^Department of Anesthesiology and Perioperative Medicine, The Second Affiliated Hospital and Yuying Children’s Hospital of Wenzhou Medical University, Wenzhou, Zhejiang, China; ^2^Key Laboratory of Pediatric Anesthesiology, Ministry of Education, Wenzhou Medical University, Wenzhou, Zhejiang, China; ^3^Key Laboratory of Anesthesiology of Zhejiang Province, Wenzhou Medical University, Wenzhou, Zhejiang, China

**Keywords:** knowledge, attitude, anesthesia, surgical patients and their families, cross-sectional study

## Abstract

**Introduction:**

Anesthesia plays a critical role in modern surgical procedures by ensuring patient pain management and safety. This study aimed to investigate the knowledge and attitude of surgical patients and their families toward anesthesia.

**Methods:**

This prospective, cross-sectional study included patients and their families in Wenzhou, China. Data collection and the measurement of knowledge and attitude scores were administered using a self-administered questionnaire.

**Results:**

503 participants (69.98% patients, 30.02% families) were included. The mean knowledge and attitude scores were 7.93 ± 6.11 (possible range: 0–26), and 32.64 ± 2.59 (possible range: 8–40), respectively, indicating an inadequate knowledge and positive attitude. Moreover, a multivariable logistic regression analysis showed that age [odd ratio (OR) = 0.394, *p* = 0.018], residence (OR = 0.424, *p* = 0.002), household income per month (OR = 0.297 ~ 0.380, *p* < 0.05), gender (OR = 1.680, *p* = 0.017), education (OR = 2.891, *p* = 0.017), and experienced anesthesia (OR = 4.405, *p* = 0.001) were independently associated with knowledge score. Additionally, knowledge score (OR = 1.096, *p* < 0.001), relationship with the patient (OR = 1.902, *p* = 0.009), and household income per month (OR = 0.545, *p* < 0.031) were independently associated with attitude score.

**Discussion:**

In conclusion, surgical patients and their families in Wenzhou, China had inadequate knowledge while positive attitude towards anesthesia, which might be influenced by their sociodemographic characteristics, including age, gender, residence, education, household income, relationship with patient, and experienced anesthesia. These findings emphasize the necessity of customized educational programs aimed at improving anesthesia knowledge and attitudes of patients and their families, especially among those with older age and lower socioeconomic status.

## 1 Introduction

Major surgeries, numbering over 300 million annually (1), rely heavily on anesthesia to ensure patient comfort, pain management, and safety (2). This involves the administration of medication to induce a controlled state of unconsciousness, eliminate pain sensations, and maintain physiological stability throughout the surgical procedure (3). Successful outcomes hinge not only on the expertise of anesthesia providers but also on patient and family cooperation, necessitating adherence to pre-anesthesia instructions and family support (4). Anesthesia is crucial for risk–benefit comprehension and active cooperation.

Preoperative education through an Anesthesia Service Platform proved effective in alleviating anxiety, improving well-being, and reducing hospital stays (5). The study by Elkassabany et al. links preoperative education to an increased preference for spinal anesthesia (6). Inadequate anesthesia knowledge breeds misconceptions, fear, and anxiety, impacting patient decisions and satisfaction (7). Therefore, assessing patient and family understanding is vital to inform targeted interventions for safe surgery and anesthesia implementation.

Applying the theory of knowledge, belief, and behavior to health-related behavioral changes reveals that acquiring knowledge, forming beliefs, and subsequent behaviors reduce stress responses, minimizing trauma and complications and enhancing rehabilitation outcomes (8). In a Ghanaian study, 62.4% were aware of anesthesia, with less than 15% understanding the role of anesthetists beyond the operating theater (9). In China, limited research exists on patients’ and families’ understanding of anesthesia and anesthesiologists. Therefore, this study aims to explore the knowledge and attitude of surgical patients and their families regarding anesthesia within 1 week of scheduled surgery in Wenzhou, China. Moreover, we hypothesize that some socioeconomic factors might influence the knowledge and attitude of surgical patients and their families about anesthesia.

## 2 Methods

### 2.1 Study design and subjects

This prospective, cross-sectional study was conducted at The Second Affiliated Hospital of Wenzhou Medical University between 1 January 2023 and 10 March 2023. This study was ethically approved by the ethics committee of the hospital with the number 2022-K-187-01, and written informed consent was obtained from the study participants. This study included patients and their families who scheduled surgery within 1 week without previous pre-anesthesia assessment provided that they were all 18 years or older. The types of surgery included vascular surgery, neck surgery, breast surgery, general surgery, urology surgery, cardiothoracic surgery, gastrointestinal surgery, anorectal surgery, ophthalmic surgery, ENT surgery, orthopedic surgery, obstetrics and gynecology surgery, and neurosurgery (cranioplasty). Participants who were unable to read and write Chinese characters, had illnesses, or lacked behavioral autonomy were excluded from this study.

### 2.2 Questionnaire development

The design of the questionnaire referred to the relevant literature (10, 11) and expert consensus, including “*Expert Consensus on Preanesthetic Visits and Evaluations*,” *“Expert Consensus on Preoperative Anti-Anxiety*,” “*Expert Consensus on the Prevention and Treatment of Systemic Toxicity of Local Anesthetics*,” “*Expert Consensus on the Prevention and Treatment of Postoperative Delirium in Adults*,” “*Expert Consensus on the Application of Acupoint Stimulation in the Perioperative Period*,” and “*Expert Consensus on the Prevention and Treatment of Complications of Peripheral Nerve Block*.” Six anesthesiologists with over 10 years of clinical anesthesia work and teaching experience revised the questionnaire content for clarity and readability. We randomly selected 32 patients or their families for a pilot test and calculated Cronbach’s α to ensure the consistency of the questionnaire scale. A pilot test involving 32 participants was performed with Cronbach’s α of 0.845.

The final version of the questionnaire was in Chinese and included 36 items. The basic information included 12 items; the knowledge dimension included 15 items, and the attitude dimension included 9 items. For statistical analysis, scores were assigned according to the options of the items: items 1 and 2 in the knowledge dimension did not distinguish from correct or incorrect, and only descriptive statistics were performed; items K3-8 were assigned scores according to the degree of understanding, with well-known a = 2 points, heard b = 1 point, and unclear c = 0 points; items K9, 10, 12, 13, and 15 with 2 points for correct (a) answers and 0 points for wrong (b) or unclear (c) answers; items K11 and 14 with 2 points for wrong (b) answers and 0 points for correct (a) or unclear answers (c). The knowledge probable score ranges from 0 to 26 points. The attitude dimension used a five-point Likert scale, items 1, 3, 4, 5, 6, and 9 ranged from very positive (5 points) to very negative (1 point) according to the degree of positivity; items 2 and 7 are opposite ranged from very positive (0 points) to very negative (5 points) according to the degree of positivity. Item A8, which investigates the reasons why patients or families feel worried about anesthesia, was not assigned a score, so the probable attitude scores range from 8 to 40 points.

### 2.3 Data collection procedure

The survey was conducted through face-to-face distribution of questionnaires by the same worker in the anesthesia outpatient clinic or ward using convenient sampling. The period from the first participant recruited to the last participant recruited was between 1 January 2023 and 10 March 2023. Investigators explain the purpose of this questionnaire survey to participants and obtain informed consent in person. After the respondents gave their informed consent, they began to fill out the paper questionnaire, and if they have any confusion about the questionnaire content, they can consult the researchers at any time. After all the data were collected, the questionnaire underwent a quality check by the team members. The data were double-entered into a Microsoft Excel spreadsheet and validated for errors. Responses were flagged as invalid if they contained obvious logical errors, such as indicating age of 5 years for an adult participant, or if they exhibited a pattern of consistently choosing the same options in any dimension of knowledge and attitude. These invalid responses were excluded from the analysis to ensure data accuracy and reliability.

### 2.4 Sample size

The sample size was calculated using the formula for cross-sectional studies: *α* = 0.05, *n* = (*Z*_(1-*α*/2)/δ)^2 × *p* × (1-*p*) where *Z*_(1-*α*/2) = 1.96 for *α* = 0.05, *p* is assumed to be 0.5 to maximize the required sample size, and δ represents the allowable error (which was set at 5% in this study). The calculated theoretical sample size was 480, which included an additional 20% to accommodate potential subjects lost during the study.

### 2.5 Statistical analysis

SPSS 22.0 (IBM Corp., Armonk, NY, USA) was used for statistical analysis. We utilized the Kolmogorov–Smirnov tests to evaluate the normal distribution of each continuous variable. The results show that the *p*-value of the normality test is less than 0.001, indicating that the normal distribution is not consistent. However, due to the relatively large sample size, the central limit theorem is complied with by default, and the statistics are carried out according to the normal distribution. The continuous variables were expressed by mean and standard deviation (SD), comparisons between two groups were performed using Student’s t-test, and comparisons between multiple groups were performed using ANOVA. The categorical variables were expressed by *n* (%). Pearson’s correlation analysis was used to analyze the correlations between each pair of knowledge and attitude scores. The independent risk factors associated with knowledge and attitude scores were analyzed using a multivariable logistic regression analysis. Knowledge and attitude scores were categorized based on the mean score, with knowledge classified as (≥8/<8) and attitude as (≥33/<33). The variables with *p* < 0.05 in the univariate logistic regression were included in the multivariable regression. Odds ratios (OR) were then calculated to quantify the likelihood of the outcome occurring in the exposed group compared with the unexposed group. An OR greater than 1 indicates a higher odd of the outcome in the exposed group, while an OR less than 1 suggests a lower odd of the outcome in the exposed group compared with the unexposed group. Those with knowledge scores equal to or greater than 70% of the possible range were categorized as having “adequate knowledge.” Similarly, individuals with attitude scores equal to or greater than 70% of the possible range were classified as having a “positive attitude.” A two-sided *p* < 0.05 was considered statistically significant.

## 3 Results

After initially collecting 504 questionnaires for the study, careful examination led to the exclusion of 1 questionnaire with invalid responses, resulting in a response rate of 99.80%. A total of 503 valid questionnaires were included, with 352 (69.98%) patients and 151 (30.02%) of their families. Among them, 249 (49.5%) were men, 274 (54.48%) were younger than 40 years old. The average knowledge and attitude scores were 7.93 ± 6.11 (possible range: 0–26) and 32.64 ± 2.59 (possible range: 8–40), respectively, indicating inadequate knowledge and positive attitude (Table 1).

**Table 1:** Baseline characteristics and scores of knowledge and attitude.

Variables	*N* (%)	Knowledge score		Attitude score	
		Mean ± SD	*p*	Mean ± SD	*p*
Total	503	7.93 ± 6.11		32.64 ± 2.59	
Patient or family			<0.001		<0.001
Patient	352 (69.98)	7.23 ± 6.08		32.18 ± 2.64	
Family	151 (30.02)	9.56 ± 5.88		33.74 ± 2.11	
Age			<0.001		<0.001
19–30	88 (17.50)	9.43 ± 5.63		33.92 ± 2.21	
31–40	186 (36.98)	9.59 ± 6.00		33.24 ± 2.47	
41–50	133 (26.44)	5.98 ± 5.76		31.87 ± 2.36	
> 51	96 (19.09)	6.03 ± 5.96		31.39 ± 2.61	
Gender			0.051		0.794
Male	249 (49.50)	7.39 ± 5.82		32.67 ± 2.59	
Female	254 (50.50)	8.46 ± 6.35		32.61 ± 2.60	
Marital status			0.158		0.002
Unmarried	65 (12.92)	7.17 ± 4.54		33.55 ± 2.28	
Married	408 (81.11)	8.17 ± 6.32		32.57 ± 2.59	
Divorced/Widowed	30 (5.96)	6.33 ± 5.96		31.70 ± 2.74	
Residence			<0.001		<0.001
Rural	161 (32.01)	6.03 ± 5.69		31.81 ± 2.44	
Urban	215 (42.74)	10.02 ± 6.04		33.39 ± 2.63	
Suburban	127 (25.25)	6.80 ± 5.66		32.43 ± 2.36	
Education			<0.001		<0.001
Primary school and below	26 (5.17)	4.23 ± 4.96		30.88 ± 2.78	
Middle school	170 (33.80)	5.41 ± 5.60		31.63 ± 2.73	
High school/technical secondary school	125 (24.85)	7.79 ± 5.96		32.80 ± 2.11	
Junior college/undergraduate	168 (33.40)	10.92 ± 5.21		33.74 ± 2.21	
Postgraduate and above	14 (2.78)	10.79 ± 8.15		33.64 ± 2.37	
Medical-related professions			0.003		0.009
Yes	4 (0.80)	17.00 ± 8.41		36.00 ± 2.16	
No	499 (99.20)	7.86 ± 6.04		32.62 ± 2.58	
Household *per capita* income, Yuan			<0.001		<0.001
<2000	43 (8.55)	3.42 ± 3.75		31.37 ± 2.94	
2000–4,999	133 (26.44)	5.02 ± 5.71		31.34 ± 2.55	
5,000–9,999	186 (36.98)	8.76 ± 5.81		33.15 ± 2.27	
10,000–19,999	86 (17.10)	10.74 ± 4.94		33.53 ± 2.33	
≥20,000	55 (10.93)	11.29 ± 6.20		33.69 ± 2.23	
Type of medical insurance			0.006		0.417
Social medical insurance only (e.g., employee medical insurance, “new cooperative,” and “urban residence medical insurance”)	440 (87.48)	7.81 ± 6.13		32.63 ± 2.62	
Commercial medical insurance only	17 (3.38)	8.24 ± 6.18		32.12 ± 2.78	
Both social and commercial medical insurance	35 (6.96)	10.63 ± 5.47		33.23 ± 2.00	
No insurance	11 (2.19)	3.64 ± 3.91		32.18 ± 2.79	
Type of anesthesia (your family) planned to use			<0.001		<0.001
General anesthesia	257 (51.09)	10.27 ± 5.84		33.14 ± 2.41	
Intraspinal anesthesia	15 (2.98)	8.13 ± 5.72		33.33 ± 2.50	
Peripheral nerve block	2 (0.40)	0		27.50 ± 0.71	
Other local anesthesia	9 (1.79)	8.44 ± 4.39		33.78 ± 2.44	
Unclear	220 (43.74)	5.23 ± 5.34		32.01 ± 2.64	
Undergone surgery			<0.001		0.244
Yes	303 (60.24)	8.94 ± 5.90		32.53 ± 2.56	
No	200 (39.76)	6.40 ± 6.10		32.81 ± 2.64	
Experienced anesthesia			<0.001		0.376
No	182 (36.18)	5.98 ± 6.11		32.78 ± 2.71	
Yes	321 (63.82)	9.03 ± 5.83		32.57 ± 2.52	
General anesthesia	139 (27.63)	11.12 ± 5.42		32.49 ± 2.38	
Intraspinal anesthesia	81 (16.10)	11.02 ± 5.57		34.33 ± 0.58	
Peripheral nerve block	3 (0.60)	15.67 ± 7.51		33.11 ± 2.37	
Other local anesthesia	44 (8.75)	10.43 ± 5.69		31.99 ± 2.80	
Have undergone anesthesia, but unclear about the type of anesthesia	84 (16.70)	4.81 ± 4.70		32.57 ± 2.52	

In the knowledge dimension, the highest number of respondents (80.72%) chose the option “Unclear” in the question “Do you know the risks of various local anesthesia.” Only 96 (19.09%) individuals selected “well known” in the question “why it is necessary to fast and abstain from food and water before general anesthesia.” The item with the highest correct rate was “All patients preparing to undergo anesthesia must be evaluated by an anesthesiologist prior to anesthesia” (74.35%). The item with the lowest correct rate was “It is normal to not wake up 2 h after general anesthesia” (13.52%) (Supplementary Table S1). Furthermore, participants who were younger than 40 years old, living in urban areas, highly educated, medical staff or students, with higher household income, and covered by both social and commercial medical insurance had higher knowledge scores (*p* < 0.05). Additionally, experienced anesthesia showed higher knowledge scores compared with others (*p* < 0.05). It should be noticed that the patients attained lower knowledge scores than their families (Table 1, *p* < 0.001). During the subgroup analysis of knowledge (Supplementary Table S2), it was observed that families scored significantly higher than the patients in eight questions assessing the level of knowledge (*p* < 0.05). The item showing the most significant difference was “The patient is conscious during local anesthesia” with a score of 1.44 ± 0.90 in families compared with 0.99 ± 1.00 in patients (*p* < 0.001).

The attitude response revealed that most participants exhibited a generally positive attitude toward anesthesia, with 81.31% thinking that anesthesia is important in the whole surgical procedure and 78.93% strongly agreeing that truthfully informing the anesthesiologists of their own allergy history to food and drugs and any of their combined systemic diseases is important (Supplementary Table S3). Participants who were 19–30 years old, unmarried, living in an urban area, with a higher level of education, medical staff, or medical students, with a higher household income, exhibited higher attitude scores compared with their counterparts (*p* < 0.05). Patients attained lower attitude scores than their families (*p* < 0.001, Table 1). Based on the subgroup analysis, it was evident that the family members achieved significantly higher scores in nearly all the attitude questions (Supplementary Table S4).

**Figure 1:**
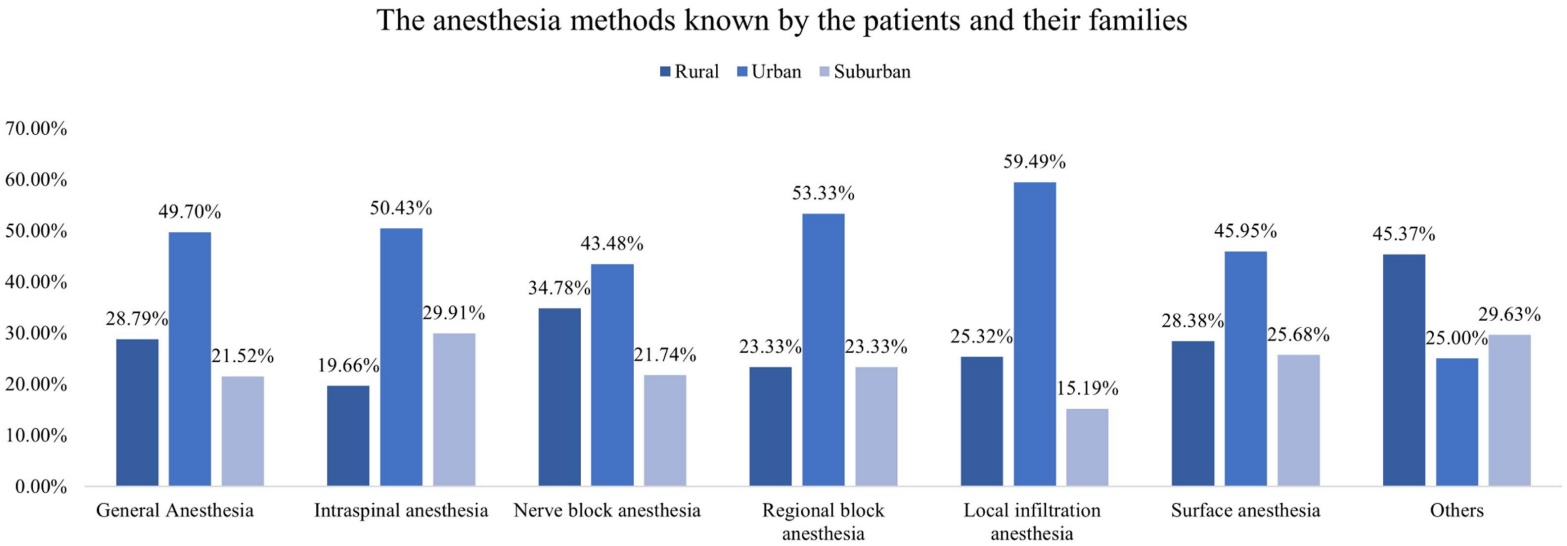
Different anesthesia methods known by the patients and their families.

**Figure 2:**
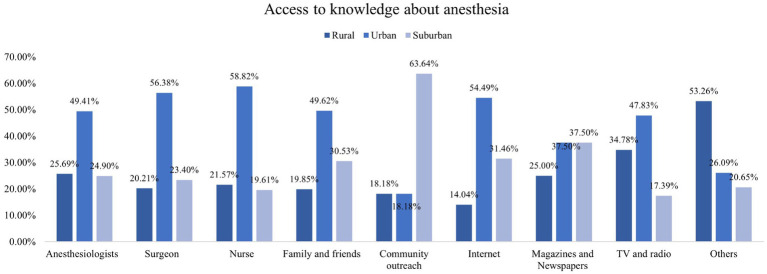
Accesses to get knowledge about anesthesia.

**Figure 3:**
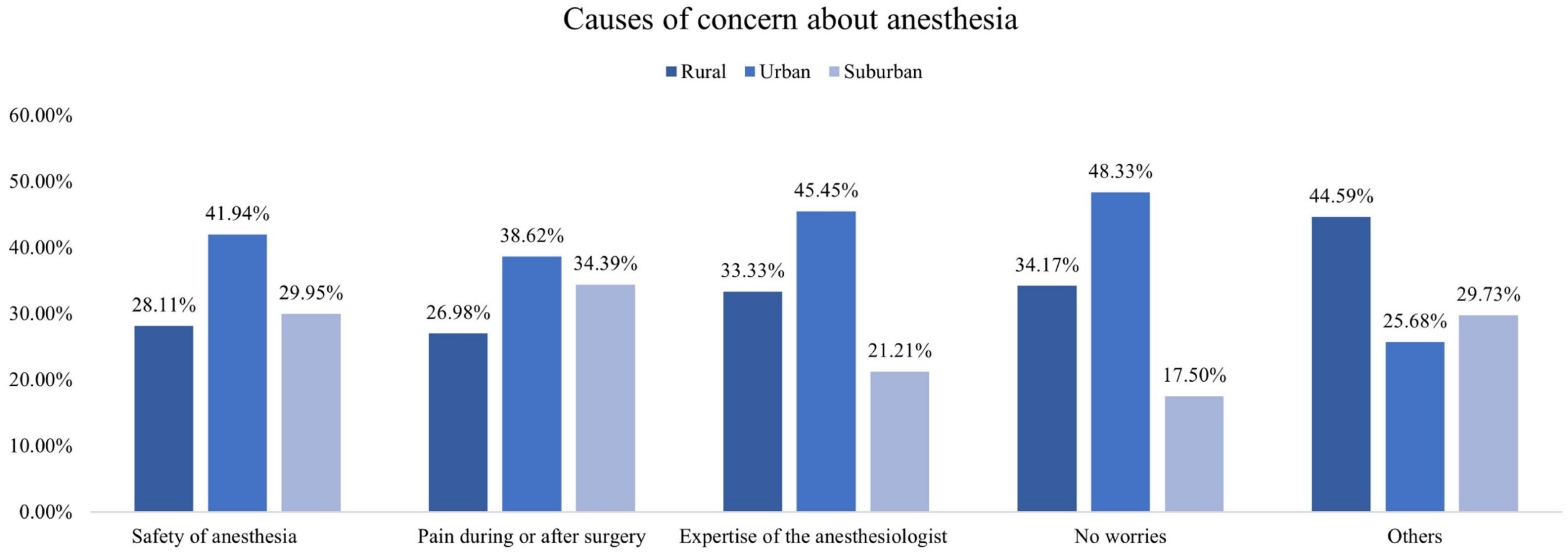
Causes of concern about anesthesia.

In addition, this study found that participants living in urban areas exhibited higher knowledge of almost all types of anesthesia methods compared with those in rural and suburban areas (Figure 1). In the access to knowledge about anesthesia, those who lived in urban areas were more likely to get knowledge from professionals (such as anesthesiologists, surgeons, and nurses) and the Internet (Figure 2). Concerning the causes of anxiety regarding anesthesia, individuals living in urban, rural, and suburban areas demonstrated comparable levels of concern, including worries about safety and pain during or after surgery and the professionalism of anesthesiologists (Figure 3). It is worth noting that among participants who chose it for other reasons, they expressed concerns that anesthesia would weaken memory or influence intelligence.

Furthermore, Pearson’s correlation analysis showed that knowledge was positively associated with attitude (*r* = 0.354, *p* < 0.001) (Table 2). Multivariable logistic regression analysis showed that participants aged 41–50 years old [odd ratio (OR) = 0.394, 95% confidence interval (CI): 0.182–0.852], living in suburban (OR = 0.424, 95% CI: 0.248–0.724), with household income less than 5,000 Yuan per month (OR = 0.297–0.380, 95% CI: 0.116–0.760) showed significantly lower odds of having adequate knowledge, while those being female (OR = 1.680, 95% CI: 1.099–2.567), with junior college/undergraduate education (OR = 2.891, 95% CI: 1.413–5.915) and experience of anesthesia (OR = 4.405, 95% CI: 1.420–13.664) showed significantly higher odds of having adequate knowledge. Moreover, participants with a higher knowledge score (OR = 1.096, 95% CI: 1.054–1.140) and being family members of the patient (OR = 1.902, 95% CI: 1.171–3.089) showed significantly higher odds of holding a positive attitude. On the other hand, those with a household income of 2000–4,999 Yuan per month (OR = 0.545, 95% CI: 0.314–0.947, *p* < 0.031) showed significantly lower odds of a holding positive attitude (Table 3).

**Table 2:** Pearson’s correlation analysis.

	Knowledge	Attitude
Knowledge	1	
Attitude	0.354 (*p* < 0.001)	1

**Table 3:** Multivariable logistic regression analysis.

	Variables			Multivariable logistic regression	
				OR (95% CI)	*p*
Knowledge	**Mean value: 7.93**	**<8, *n* (%)**	**≥8, *n* (%)**		
		257 (51.09)	246 (48.91)		
	Age				
	19–30	37 (14.40)	51 (20.73)	Ref.	
	31–40	72 (28.02)	114 (46.34)	0.852 (0.440 1.650)	0.635
	41–50	86 (33.46)	47 (19.11)	0.394 (0.182 0.852)	0.018
	>51	62 (24.12)	34 (13.82)	0.740 (0.320 1.710)	0.481
	Gender				
	Male	141 (54.86)	108 (43.90)	Ref.	
	Female	116 (45.14)	138 (56.10)	1.680 (1.099 2.567)	0.017
	Residence				
	Urban	104 (40.47)	57 (23.17)	Ref.	
	Rural	75 (29.18)	140 (56.91)	0.839 (0.475 1.481)	0.544
	Suburban	78 (30.35)	49 (19.92)	0.424 (0.248 0.724)	0.002
	Education				
	Primary school and below	21 (8.17)	5 (2.03)	0.432 (0.130 1.436)	0.171
	Middle school	118 (45.91)	52 (21.14)	Ref.	
	High school/technical secondary school	63 (24.51)	62 (25.20)	1.766 (0.967 3.228)	0.064
	Junior college/undergraduate	47 (18.29)	121 (49.19)	2.891 (1.413 5.915)	0.004
	Postgraduate and above	8 (3.11)	6 (2.44)	1.069 (0.260 4.394)	0.927
	Household *per capita* income, Yuan				
	<2000	35 (13.62)	8 (3.25)	0.297 (0.116 0.760)	0.011
	2000–4,999	98 (38.13)	35 (14.23)	0.380 (0.214 0.675)	0.001
	5,000–9,999	83 (32.30)	103 (41.87)	Ref.	
	10,000–19,999	26 (10.12)	60 (24.39)	1.198 (0.616 2.329)	0.595
	≥20,000	15 (5.84)	40 (16.26)	1.958 (0.873 4.393)	0.103
	Patient or family				
	Patient	198 (77.04)	154 (62.60)	Ref.	
	Families	59 (22.96)	92 (37.40)	1.149 (0.697 1.894)	0.585
	Undergone surgery				
	No	87 (39.01)	113 (40.36)	Ref.	
	Yes	136 (60.99)	167 (59.64)	1.027 (0.336 3.138)	0.963
	Undergone anesthesia				
	No			Ref.	
	Yes			4.405 (1.420 13.664)	0.010
Attitude	**Mean value: 32.64**	**<33, *n* (%)**	**≥33, *n* (%)**		
		223 (44.33)	280 (55.67)		
	Knowledge score			1.096 (1.054 1.140)	<0.001
	Age				
	19–30	26 (11.66)	62 (22.14)		
	31–40	57 (25.56)	129 (46.07)	0.936 (0.499 1.757)	0.837
	41–50	77 (34.53)	56 (20.00)	0.500 (0.242 1.033)	0.061
	>51	63 (28.25)	33 (11.79)	0.458 (0.207 1.011)	0.053
	Residence				
	Urban	91 (40.81)	70 (25.00)	Ref.	
	Rural	66 (29.60)	149 (53.21)	0.854 (0.491 1.486)	0.576
	Suburban	66 (29.60)	61 (21.79)	0.630 (0.375 1.060)	0.082
	Education				
	Primary school and below	21 (8.17)	5 (2.03)	0.722 (0.257 2.031)	0.538
	Middle school	118 (45.91)	52 (21.14)	Ref.	
	High school/technical secondary school	63 (24.51)	62 (25.20)	1.365 (0.757 2.463)	0.301
	Junior college/undergraduate	47 (18.29)	121 (49.19)	1.184 (0.583 2.405)	0.640
	Postgraduate and above	8 (3.11)	6 (2.44)	1.221 (0.293 5.095)	0.784
	Household *per capita* income, Yuan				
	<2000	27 (12.11)	16 (5.71)	1.045 (0.468 2.333)	0.915
	2000–4,999	89 (39.91)	44 (15.71)	0.545 (0.314 0.947)	0.031
	5,000–9,999	70 (31.39)	116 (41.43)	Ref.	
	10,000–19,999	27 (12.11)	59 (21.07)	0.928 (0.494 1.744)	0.817
	≥20,000	10 (4.48)	45 (16.07)	1.950 (0.324 0.854)	0.120
	Patient or family				
	Patient	184 (82.51)	168 (60.00)	Ref.	
	Families	39 (17.49)	112 (40.00)	1.902 (1.171 3.089)	0.009

Knowledge score <8 and ≥8 means inadequate and adequate knowledge, respectively; Attitude score <33 and ≥33 means negative and positive attitude, respectively.

## 4 Discussion

This study aimed to explore the knowledge and attitudes of surgical patients and their families toward anesthesia. The findings revealed that surgical patients and their families had inadequate knowledge but positive attitudes toward anesthesia, indicating a need for the popularization and promotion of anesthesia-related knowledge. Factors such as age, gender, residence, education, household income, and experienced anesthesia were found to be associated with participants’ cognition of anesthesia. The findings might be beneficial for the need for targeted education and promotion programs to enhance anesthesia management.

In this study, the average knowledge scores among the participants were relatively low. These results are consistent with a study conducted in Ethiopia (12), despite the considerable differences in terms of economic status, medical facilities, and medical education between the two countries. The Ethiopian study reported that 71.7% of participants had a poor level of knowledge of anesthesia, answering less than half of the questions correctly (12). The consistent findings across different regions highlight the need for widespread efforts to improve patient education and awareness about anesthesia. Therefore, it is crucial to enhance the education of patients and their families in anesthesia. By offering clear and precise information about anesthesia, including its purpose, risks, and benefits, anesthesiologists can reduce preoperative anxiety and positively influence postoperative outcomes (13).

Further investigation revealed that several factors were associated with the knowledge and attitude regarding anesthesia. Specifically, having a junior college or undergraduate education level emerged as an independent factor associated with good knowledge. This finding aligns with a study in India, which also demonstrated that higher education levels contribute to greater knowledge about anesthesia among the general population (14). However, a previous study also showed that despite participants’ high level of education, their health literacy of the roles of anesthesiologists was also limited (15). This discrepancy might be attributed to the fact that the focus of this study was on personal health management related to anesthesia rather than specifically assessing knowledge about the roles of anesthesiologists. Moreover, no significant findings were observed among participants with a postgraduate education level and above likely due to the small sample size in this category.

Experience with anesthesia often involves interaction with healthcare professionals and educational materials, which can contribute to a better understanding of anesthesia-related concepts. We found that individuals who had previously experienced anesthesia showed higher knowledge scores. Familiarity with the healthcare process may result in increased knowledge scores among those with previous exposure to anesthesia. It suggests that exposure to anesthesia contributes to a better understanding of anesthesia-related concepts. However, this finding is inconsistent with a previous study in India, where participants had limited knowledge about anesthesia, despite the majority of them (62%) having undergone previous surgery (16). This may be because the factor of having surgery did not demonstrate an independent association. One possible explanation is that undergoing surgery itself may not directly influence an individual’s knowledge of anesthesia. Additionally, the complexity and diversity of surgical procedures may lead to varying experiences that do not uniformly impact knowledge scores. These findings highlight the importance of targeted educational interventions and communication strategies in enhancing patients’ knowledge of anesthesia, particularly among those without prior anesthesia experience.

Additionally, we observed that 51.09% of patients preferred general anesthesia, while 43.74% were undecided or unclear about their anesthesia choice. Other types of anesthesia, such as intraspinal anesthesia, peripheral nerve block, and other local anesthesia, had relatively low percentages. However, logistic regression analysis did not reveal any significant differences in knowledge and attitudes based on the type of anesthesia chosen. This may be because the patient’s knowledge is insufficient, so their choice of the type of anesthesia is more likely to depend on the doctor’s decision. Furthermore, it was surprising to note that in the subgroup analysis of knowledge, families scored significantly higher than patients in the majority of questions (8/14). The observed disparity between surgical patients and their families regarding anesthesia is a multifaceted issue with potential implications for patient outcomes and healthcare communication. The differences in information dissemination can be crucial, with healthcare providers, often prioritizing families as key decision-makers and thus providing them with more detailed explanations during pre-operative consultations (17). Moreover, patients facing surgery may experience heightened levels of anxiety and stress, potentially impairing their ability to fully grasp the information provided. The reported incidence of preoperative anxiety ranges from 60 to 92% in patients (18). To bridge this knowledge gap, healthcare providers should prioritize clear, patient-centered communication, tailored to individual needs, to ensure that both patients and their families are well-informed and actively engaged in decisions related to anesthesia (19).

The findings of this study shed light on the disparities in knowledge and sources of information regarding anesthesia based on their residential areas. The observed discrepancy between urban and rural/suburban participants in terms of anesthesia knowledge is of significant concern. The findings of this study align with previous research on healthcare disparities in urban and rural areas, which has shown discrepancies in access to healthcare resources and information (12, 20). The urban population exhibited a notably higher level of awareness about various anesthesia methods and were more likely to acquire knowledge from professionals as well as the Internet. In contrast, those from rural and suburban areas may have less access to these sources, emphasizing the importance of developing alternative avenues for education in these regions. It underscores the need for targeted educational campaigns and improved dissemination of information in rural and suburban areas to bridge this knowledge gap. Moreover, the study highlighted that regardless of their location, participants shared similar concerns about anesthesia, which is similar to the previous study (21). Therefore, it is crucial for healthcare providers to address these common anxieties through pre-operative counseling and patient education.

The limitations of this study should be acknowledged. First, the study was conducted at a single hospital, which may limit the generalizability of the findings. Second, the reliance on self-reported data through questionnaires introduces the possibility of recall bias or social desirability bias. Additionally, the cross-sectional design of the study prevents the establishment of causal relationships and the assessment of temporal changes in knowledge and attitude. Longitudinal studies would be beneficial in capturing changes in anesthesia knowledge and attitude over time.

In conclusion, surgical patients and their families in Wenzhou, China, had inadequate knowledge while a positive attitude toward anesthesia might be influenced by their sociodemographic characteristics, such as age, gender, residence, education, household income, relationship with the patient, and prior experience with anesthesia. These findings emphasize the necessity of customized educational programs aimed at improving anesthesia knowledge and attitudes for patients and their families, especially among individuals from older age groups and those with lower socioeconomic status.

## Data availability statement

The original contributions presented in the study are included in the article/Supplementary material, further inquiries can be directed to the corresponding author.

## Ethics statement

The studies involving humans were approved by Medical Ethics Committee of the Second Affiliated Hospital and Yuying Children’s Hospital of Wenzhou Medical University (No. 2022-K-187-01). The studies were conducted in accordance with the local legislation and institutional requirements. The participants provided their written informed consent to participate in this study.

## Author contributions

JW: Conceptualization, Data curation, Formal analysis, Investigation, Writing – original draft, Writing – review & editing. SW: Data curation, Formal analysis, Investigation, Writing – original draft, Writing – review & editing. RZ: Conceptualization, Data curation, Formal analysis, Investigation, Writing – original draft, Writing – review & editing.
