# Supplementary material for: Knowledge and attitude of surgical patients and their families toward anesthesia
Source: Front Med (Lausanne). 2024 May 22;11:1371785. doi: 10.3389/fmed.2024.1371785 (PMC11150525; doi:10.3389/fmed.2024.1371785)
Supplement: Supplementary file 1 [file Table_1.DOCX]

**Supplementary materials**

**Knowledge and attitude of** **surgical patients and their families towards** **anesthesia**

**Running title**: K & A on anesthesia

Jie Wang, MS, Shuai Wang, MS, Ruifeng Zeng, PhD*

Department of Anesthesiology and Perioperative Medicine, The Second Affiliated Hospital and Yuying Children’s Hospital of Wenzhou Medical University; key Laboratory of Pediatric Anesthesiology, Ministry of Education, Wenzhou Medical University; key Laboratory of Anesthesiology of Zhejiang Province, Wenzhou Medical University.

***Corresponding authors:**

Ruifeng Zeng

Tel: + (86) 13857734776

E-mail: ruifengzengwmu@163.com

**Table S1 Knowledge dimension**

| **Items** | **Well known, n (%)** | **Heard, n (%)** | **Unclear, n (%)** |
| --- | --- | --- | --- |
| **3. Do you know the routes of drug delivery for general anesthesia?** | 54 (10.74) | 115 (22.86) | 334 (66.40) |
| **4. Do you know why it is necessary to fast and abstain from food and water before general anesthesia?** | 96 (19.09) | 110 (21.87) | 297 (59.05) |
| **5. Do you know the risks of general anesthesia?** | 35 (6.96) | 167 (33.20) | 301 (59.84) |
| **6. Do you know the risks of various local anesthesia?** | 21 (4.17) | 76 (15.11) | 406 (80.72) |
| **7. Do you know the position of intraspinal anesthesia?** | 78 (15.51) | 38 (7.55) | 387 (76.94) |
| 1. **Do you know the purpose of anesthesia clinic consultation and preoperative visit for anesthesia?** | 30 (5.96) | 107(21.27) | 366(72.76) |
|  | **Correct** | **Wrong** | **Unclear** |
| **9. All patients preparing to undergo anesthesia must be evaluated by an anesthesiologist prior to anesthesia.** | 374 (74.35) | 3 (0.60) | 126 (25.05) |
| **10. The patient is conscious during local anesthesia.** | 284 (56.46) | 18 (3.58) | 201 (39.96) |
| **11.** **Local anesthetics do not cause systemic toxic reactions.** | 48 (9.54) | 113 (22.57) | 342 (67.99) |
| **12. In the state of general anesthesia, the patient is usually unable to breathe on his/her own because of unconsciousness and general muscle relaxation, and therefore requires tracheal intubation for oxygen delivery.** | 150 (29.82) | 39 (7.75) | 314 (62.43) |
| **13. During general anesthesia, the anesthesiologist will perform real-time monitoring to keep an eye on the vital signs of patients.** | 275 (54.67) | 2 (0.40) | 226 (44.93) |
| **14. It is normal to not wake up two hours after general anesthesia.** | 72 (14.31) | 68 (13.52) | 363 (72.17) |
| **15. Postoperative nausea and vomiting are one of the most common postoperative adverse reactions for anesthesia.** | 171 (34.00) | 15 (2.98) | 317 (63.02) |

**Table S2 Subgroup analysis of knowledge dimension**

| **Items** | **Score** | | **P** |
| --- | --- | --- | --- |
|  | **Patients** | **Families** |  |
| **3. Do you know the routes of drug delivery for general anesthesia?** | 0.44 ± 0.69 | 0.44 ± 0.67 | 0.994 |
| **4. Do you know why it is necessary to fast and abstain from food and water before general anesthesia?** | 0.54 ± 0.77 | 0.74 ± 0.81 | 0.008 |
| **5. Do you know the risks of general anesthesia?** | 0.46 ± 0.62 | 0.50 ± 0.63 | 0.548 |
| **6. Do you know the risks of various local anesthesia?** | 0.23 ± 0.51 | 0.25 ± 0.52 | 0.626 |
| **7. Do you know the position of intraspinal anesthesia?** | 0.30 ± 0.66 | 0.58 ± 0.88 | <0.001 |
| **8. Do you know the purpose of anesthesia clinic consultation and preoperative visit for anesthesia?** | 0.30 ± 0.56 | 0.42 ± 0.64 | 0.032 |
| **9. All patients preparing to undergo anesthesia must be evaluated by an anesthesiologist prior to anesthesia.** | 1.39 ± 0.92 | 1.72 ± 0.69 | <0.001 |
| **10.** **The patient is conscious during local anesthesia.** | 0.99 ± 1.00 | 1.44 ± 0.90 | <0.001 |
| **11. Local anesthetics do not cause systemic toxic reactions.** | 0.18 ± 0.58 | 0.21 ± 0.62 | 0.599 |
| **12. In the state of general anesthesia, the patient is usually unable to breathe on his/her own because of unconsciousness and general muscle relaxation, and therefore requires tracheal intubation for oxygen delivery.** | 0.49 ± 0.86 | 0.85 ± 0.99 | <0.001 |
| **13. During general anesthesia, the anesthesiologist will perform real-time monitoring to keep an eye on the vital signs of patients.** | 1.02 ± 1.00 | 1.27 ± 0.97 | 0.009 |
| **14. It is normal to not wake up two hours after general anesthesia.** | 0.24 ± 0.65 | 0.40 ± 0.80 | 0.020 |
| **15. Postoperative nausea and vomiting are one of the most common postoperative adverse reactions for anesthesia.** | 0.65 ± 0.94 | 0.74 ± 0.97 | 0.339 |

Table S3 Attitude dimension

| **Items** | **Strongly agree/Very important, n (%)** | **Relatively agree/ Relatively important, n (%)** | **Neutral, n (%)** | **Relatively disagree/ Relatively unimportant, n (%)** | **Strongly disagree/Very unimportant, n (%)** | **Score, mean** ± **SD** |
| --- | --- | --- | --- | --- | --- | --- |
| **1. The anesthesia is important throughout the entire surgical procure.** | 409 (81.31) | 89 (17.69) | 4 (0.80) | 1 (0.20) | 0 | 4.80 ± 0.43 |
| **2. The main role of the anesthesiologist during surgery is to reduce the patient's pain and allow the patient to sleep.** | 147 (29.22) | 228 (45.33) | 73 (14.51) | 49 (9.74) | 6 (1.19) | 2.08 ± 0.96 |
| **3. The anesthesiologist needs to monitor vital signs during surgery to protect the patient's life safety and provide conditions for surgical operations.** | 397 (78.93) | 91 (18.09) | 12 (2.39) | 3 (0.60) | 0 | 4.75 ± 0.52 |
| **4. It is important of following the medical advice to fast and abstain from food and water before surgery.** | 362 (71.97) | 138 (27.44) | 1 (0.20) | 2 (0.40) | 0 | 4.71 ± 0.48 |
| **5. It is important to truthfully inform the anesthesiologist of their own allergy history to food and drugs and any of their combined systemic diseases.** | 370 (73.56) | 130 (25.84) | 2 (0.40) | 1 (0.20) | 0 | 4.73 ± 0.47 |
| **6. It is important to keep a good emotional state before surgery.** | 350 (69.58) | 146 (29.03) | 6 (1.19) | 1 (0.20) | 0 | 4.68 ± 0.50 |
| **7. Do you feel afraid or worried about anesthesia?** | 86 (17.10) | 187 (37.18) | 139 (27.63) | 69 (13.72) | 22 (4.37) | 2.51 ± 1.06 |
| **9. Do you wish to learn more about anesthesia-related knowledge?** | 280 (55.67) | 150 (29.82) | 59 (11.73) | 11 (2.19) | 3 (0.60) | 4.38 ± 0.82 |

**Table S4 Subgroup analysis of attitude dimension**

| **Items** | **Score** | | **P** |
| --- | --- | --- | --- |
|  | **Patients** | **Families** |  |
| **1. The anesthesia is important throughout the entire surgical procure.** | 4.76 ± 0.47 | 4.89 ± 0.33 | 0.002 |
| **2. The main role of the anesthesiologist during surgery is to reduce the patient's pain and allow the patient to sleep.** | 2.00 ± 0.89 | 2.28 ± 1.09 | 0.002 |
| **3. The anesthesiologist needs to monitor vital signs during surgery to protect the patient's life safety and provide conditions for surgical operations.** | 4.70 ± 0.57 | 4.89 ± 0.34 | <0.001 |
| **4. It is important of following the medical advice to fast and abstain from food and water before surgery.** | 4.64 ± 0.52 | 4.86 ± 0.35 | <0.001 |
| **5. It is important to truthfully inform the anesthesiologist of their own allergy history to food and drugs and any of their combined systemic diseases.** | 4.69 ± 0.49 | 4.81± 0.39 | 0.006 |
| **6. It is important to keep a good emotional state before surgery.** | 4.63 ± 0.54 | 4.81 ± 0.40 | <0.001 |
| **7. Do you feel afraid or worried about anesthesia?** | 2.46 ± 1.03 | 2.64 ± 1.14 | 0.085 |
| **9. Do you wish to learn more about anesthesia-related knowledge?** | 4.30 ± 0.87 | 4.55 ± 0.65 | 0.002 |
